# Prolongation of Electrocardiographic T Wave Parameters Recorded during the Head-Up Tilt Table Test as Independent Markers of Syncope Severity in Children

**DOI:** 10.3390/ijerph17186441

**Published:** 2020-09-04

**Authors:** Grażyna Markiewicz-Łoskot, Ewelina Kolarczyk, Bogusław Mazurek, Marianna Łoskot, Lesław Szydłowski

**Affiliations:** 1 Department of Nursing and Social Medical Problems, Faculty of Health Sciences, Medical University of Silesia in Katowice, 40-752 Katowice, Poland; mic54@o2.pl; 2 Department of Propaedeutics of Nursing, Faculty of Health Sciences, Medical University of Silesia in Katowice, 40-752 Katowice, Poland; ewelinakol@tlen.pl; 3 Department of Pediatric Cardiology, Faculty of Medical Sciences, Medical University of Silesia in Katowice, 40-752 Katowice, Poland; mazurek30@op.pl; 4 Students’ Research Group, Department of Nursing and Social Medical Problems, Faculty of Health Sciences, Medical University of Silesia in Katowice, 40-752 Katowice, Poland; marianna.loskot@gmail.com

**Keywords:** children, vasovagal syncope, long QT syndrome, head-up tilt-table test, electrocardiography, repolarization parameters, QTc and TpTe intervals

## Abstract

The head-up tilt table test (HUTT) with the upright phase is used to help determine an imbalance of the sympathetic nervous system that is related to abnormal electrocardiographic repolarization in children with vasovagal syncope (VVS) and also in patients with the long QT syndrome (LQTS). The study attempted to evaluate T wave morphology and QT and TpTe (Tpeak–Tend) intervals recorded in ECG during the HUTT for a more accurate diagnosis of children with VVS. The group investigated 70 children with a negative HUTT result: 40 patients with VVS and 30 healthy volunteers without syncope. The RR interval as well as TpTe, and QTc intervals were measured in lead V5 of electrocardiogram (ECG) on admission to the hospital and during three phases of the HUTT. In syncopal children, which included 23 children with bifid or flat T waves and 17 patients with normal T waves in the upright phase, the QTc and TpTe were longer (*p* < 0.001) compared to the other test phases and longer (*p* < 0.001) than in the control group, respectively, with the risk of arrhythmias. Only in the control group, the TpTe was shorter (*p* < 0.001) in the upright phase than in the other tilt phases. The TpTe in the upright phase (>70 ms) was a good discriminator, and was better than the QTc (>427 ms). Prolongation of electrocardiographic TpTe and QT intervals, in addition to the (abnormal T wave morphology recorded during the HUTT, are helpful for identifying VVS children more predisposed to ventricular arrhythmias with a latent risk of LQTS. Further studies are required to assess the value of these repolarization parameters in clinical practice.

## 1. Introduction

Syncope was defined as a sudden, transient loss of consciousness (T-LOC) and muscle tone due to temporal cerebral hypoperfusion (caused by episodic hypotension and/or bradycardia) characterized by a rapid onset, short duration, and spontaneous complete recovery [1]. Syncope is a frequent and serious problem in daily pediatric practice. For instance, 30% to 50% of children and adolescents aged 8 to 18 years old experience one or more syncopal episodes during their life with females showing more than twice the incidence rate than males [2,3]. A vast majority of episodes (33% to 80%) are of a benign nature and they are cases of neurally-mediated (neurocardiogenic) vasovagal syncope (VVS) occurring among children with a normal heart structure but impaired autoregulation of the cardiovascular system in response to venous pooling and catecholamine-induced tachycardia [3]. VVS is associated with reflex vasodepressor hypotension and/or bradycardia, which may be caused by prolonged verticalization and/or a sudden change of a body position with an upright posture (standing or sitting), or may occur due to exposure to emotional stress, fear, pain, or medical settings (during blood sampling) [2]. VVS is often followed by fatigue and it is frequently preceded by characteristic prodromal symptoms such as dizziness, warmth, nausea, and pallor, visual, and hearing disturbances [1]. Types of vasovagal syncope (vasodepressor, cardio-depressive, and mixed) are determined by a positive result (with development of reflex hypotension and/or bradycardia) of the head-up tilt test (HUTT) [4,5].

The diagnosis is primarily based on a careful personal and family history of syncope and/or aborted sudden cardiac death (SCD), normal physical examination, and 12-lead electrocardiogram (ECG), which can lead to a syncope diagnosis in more than approximately 50% to 60% of patients [3]. Although most cases of syncope show a benign nature, there is a small subset of children for whom a syncopal episode may herald potentially life-threatening ventricular arrhythmias associated with inherited ion channelopathies: long QT syndrome (LQTS), short QT syndrome (SQTS), Brugada syndrome, and catecholaminergic polymorphic ventricular tachyarrhythmias (CPVT) [6]. Cardiac syncope represents 1.5% to 6% of pediatric cases [3]. The presence of cardiac disease (structural dysfunction or inherited channelopathies) and abnormal ECG are predictors of cardiac arrhythmias [1]. In clinical diagnostics, abnormal electrocardiograms, particularly electrocardiographic repolarization abnormalities with QT and TpTe (Tpeak–Tend) intervals prolongation, are some of the key features used to evaluate the patient and assess the risk of malignant ventricular arrhythmias. T wave parameters: QT and, especially, the T peak to T end interval (TpTe) reflect dispersion of repolarization. Its amplification may lead to development of life-threatening ventricular arrhythmias and syncope [7,8,9]. The study attempted to evaluate T wave morphology, QT, and TpTe intervals, recorded during the head-up tilt table test, for a more accurate diagnosis of children with vasovagal syncope (VVS) and negative results of the HUTT.

## 2. Materials and Methods

The study was approved by the Bioethics Committee at the Medical University of Silesia in Katowice (No. KNW/0022/KB1/79/I/17).

All the study children were admitted to the Department of Pediatric Cardiology (Medical University of Silesia) at the John Paul II Upper Silesian Child Health Centre in Katowice (Poland). The children were strictly enrolled in the study and control groups. In the Department of Pediatric Cardiology, based on 820 medical hospitalization records referring to four years, we found 110 children with clinically diagnosed VVS, including 40 children with negative HUTT who were enrolled in the study group.

### 2.1. The Study Subjects

40 children (27 girls and 13 boys, median age 15.8 years with clinically diagnosed VVS (syncope caused by prolonged verticalization or a sudden change of the body position and in a setting of emotional stress preceded by characteristic prodromal symptoms) and a negative HUTT (without development of reflex hypotension and/or reflex bradycardia) and 30 healthy volunteers (12 girls and 18 boys, median age 16.1 years without syncope and also with negative results of HUTT were enrolled in the study. There were no differences between groups regarding age (*p* = 0.606) but there were significant differences (*p* = 0.030) in sex distribution between syncopal and control groups.

None of the 70 children had a positive personal or family history of syncope or arrhythmia, sudden cardiac death, or structural heart disease in physical examination (also neurological) and in echocardiography using the Doppler method as well as no cardiac arrhythmias in ECGs with Holter monitoring or during the exercise stress (Bruce protocol).

The study exclusion criteria were as follows: a structural heart disease, bundle branch block, or any other intraventricular conduction defect, atrial fibrillation, lack of sinus rhythm, electrolyte imbalance (K, Ca, Mg), and the use of medications known to prolong the QT interval.

#### 2.1.1. Protocol of Head-Up Tilt Table Test

HUTT performed using the Westminster Protocol, without pharmacological provocation [4]. The children were prepared for the study tilt table testing on an electronic motorized tilt table equipped with a footrest in a quiet room. They were in the fasting state. After 30 min of supine resting, the children were abruptly tilted upright to 60° for another 45 min on a tilt table with a footrest and then they returned to the supine position.

Blood pressure (BP) (Sphingomanometer 767 WelchAllyn, Hillrom, Chicago, IL, USA) and heart rate (HR) (DINAMAP PRO 1000V3, GE Medical Systems, Milwaukee, WI, USA) were measured during the pre-upright and post-upright phases as well as every 5 min following the start of the upright phase and after returning to the supine position.

#### 2.1.2. Electrocardiography

In the study, digital 12-lead resting electrocardiograms during HUTT were recorded at a paper speed of 50 mm/s, with an amplification of 10 mm/mV (As CARD MrGrey, with sampling frequency of 1500 Hz and calibration of 1mV). The ECGs were recorded on admission to hospital, on the first day of hospitalization (ECG 0), and during 3 phases of the tilt test: supine resting for 30 min (ECG 1), followed by tilting upright to 60° for another 45 min on a tilt table (ECG 2), and then returning to the supine position (ECG 3).

**Figure 1:**
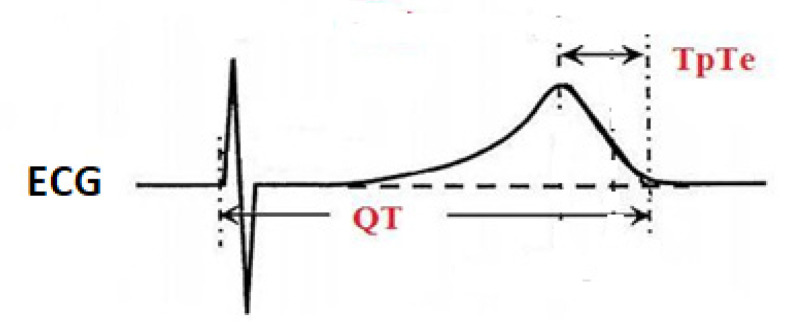
Method of measuring QT and TpTe intervals in ECG. QT—the total repolarization period, TpTe (Tpeak–Tend)—the late repolarization period. Authors’ source.

The RR interval, QT (the total repolarization period), and TpTe (the late repolarization period) intervals were measured manually in the ECG V5 precordial lead for three consecutive cardiac cycles and then averaged. The QT interval was measured from the onset of Q wave to the end of T wave at the point of its return to the isoelectric line. The end of T wave was defined as the intersection between the line tangent to the descending arm of T wave and the isoelectric line. TpTe (Tpeak-Tend) was the interval from the peak of T wave to the end of T wave at the point of its return to the isoelectric line. For the inverted T wave, the TpTe was measured from the lowest point of inverted T wave to the end of the T wave at the point of its return to the isoelectric line. The U wave was not taken into account [10] (Figure 1).

To correct for possible heart rate effects on the QT, we applied the Bazett’s (QT/√RR) formula. All measurements of the end of the T wave were analyzed blindly by another independent investigator without access to results obtained and clinical data. The highest quality ECGs were analyzed without network disturbances.

### 2.2. Statistical Analysis

The data were exported from an Excel v.2010 (Microsoft Corporation, Redmond, WA, USA) datasheet to the Statistica v.7.1 data analysis software system (StatSoft Inc., Tulsa, OK, USA) and MedCalc statistical Software v.19.07 (MedCalc Software Ltd, Ostend, Belgium).

Due to the lack of normal distribution of the investigated parameters (examined with the Shapiro-Wilk test), we used the Mann-Whitney U test with the Marascuillo and McSweeney continuity correction and Fisher’s exact test for sex differences. In addition, the ANOVA Friedman test with the Iman-Davenport statistic and post hoc test (Conover-Iman) were used for inter-time comparisons (ranges). The level for statistical significance was set at a *p* value < 0.05. The ROC curve receiver operating characteristic (area under curve—AUC) analysis and pairwise comparison of ROC curves were also performed to determine the cut-off points for TpTe (Tpeak–Tend) and QTc intervals with the highest sensitivity and specificity.

## 3. Results

The study group of 40 children with diagnosed VVS had had episodes of fainting over the period of 3 months (10/40 or 25%) up to 4 years (10/40 or 25%) before hospitalization and experienced syncope at least 3 to 4 times within the previous year. The first episode of syncope occurred at the age of 15 (2.3) years. In medical (personal) history, syncope was caused by prolonged verticalization and/or a sudden change of the body position (23/40 or 57.5%) and in a setting of emotional stress, fear, pain, or during blood sampling (16/40 or 40%). In addition, 45% (18/40) of these patients also experienced episodes of syncope post-exercise. These episodes were not accompanied by focal neurological symptoms.

In 16 children (40% of the study group), syncope was followed by fatigue and preceded by characteristic prodromal symptoms such as dizziness (14/40 or 35%), headache (15/40 or 37.5%), breathlessness (12/40 or 30%), visual disturbances (11/40 or 27.5%), tremors in the limbs (6/40 or 15%), tinnitus (4/40 or 10%), pallor (3/40 or 7.5%), sweating (12/40 or 5%), and a hot feeling (1/40 or 2.5%). Preceding palpitations in 22.5% (9/40) of patients and chest pain (6/40 or 15%) were not usual symptoms heralding VVS (Table 1).

A comparison of electrocardiographic parameters [ms] during the tilt table test between the children diagnosed with VVS (Syncopal group) and healthy volunteers without syncope (Control group) is shown in Table 2.

Based on the ECG analysis of the repolarization period, i.e., morphology and amplitude of the T-wave in ECG during the upright phase of the tilt testing, the study group consisted of 40 syncopal children, including 23 children with T wave morphology abnormality: bifid and/or flat (low amplitude) T waves and 17 children with a normal T wave.

**Figure 2:**
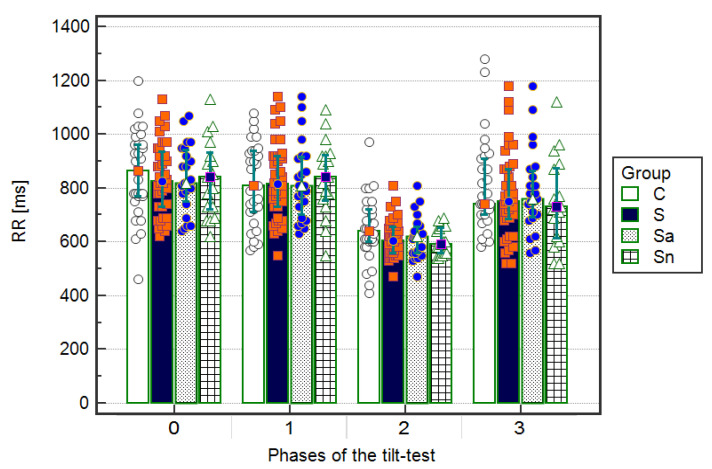
Inter-time comparison of RR intervals in electrocardiograms (ECGs) on admission to the hospital (Phase 0), during the resting supine phase (Phase 1), the upright phase (Phase 2), and the returning to supine phase (Phase 3) in the groups: C—control, S—syncopal Sa—syncopal with abnormal T, Sn—syncopal with normal T. Values [ms] are shown as a median and interquartile range. A significant difference (*p* < 0.05) was calculated using the ANOVA Friedman test with the Iman-Davenport statistic and post hoc test (Conover-Iman).

The RR intervals, shown as median and IQR (interquartile range) [ms], were significantly (*p* < 0.001) shorter in the upright phase (RR2) compared to ECGs on admission to the hospital (RR0) during the supine resting (RR1) and after returning to a supine position (RR3) in the whole group of 40 children with syncope: median 605 ms (IQR 95 ms) vs. median 825 ms (IQR 205 ms) and median 815 ms (IQR 190 ms) and median 750 ms (IQR 185 ms), respectively. Afterward, after dividing to subgroups, 23 children with an abnormal T wave: 620 (100) vs. 820 (210) and 810 (230) and 760 (180), respectively, and 17 children without changes in the repolarization period: 590 (90) vs. 840 (190) and 840 (160) and 730 (250), respectively, as well as among 30 children in the control group: 640 (120) vs. 865 (190) and 810 (230) and 740 (210), respectively. There were no significant differences between the four groups in the RR intervals during ECG on admission and during the three phases of head-up tilt testing (Figure 2, Table 3).

**Figure 3:**
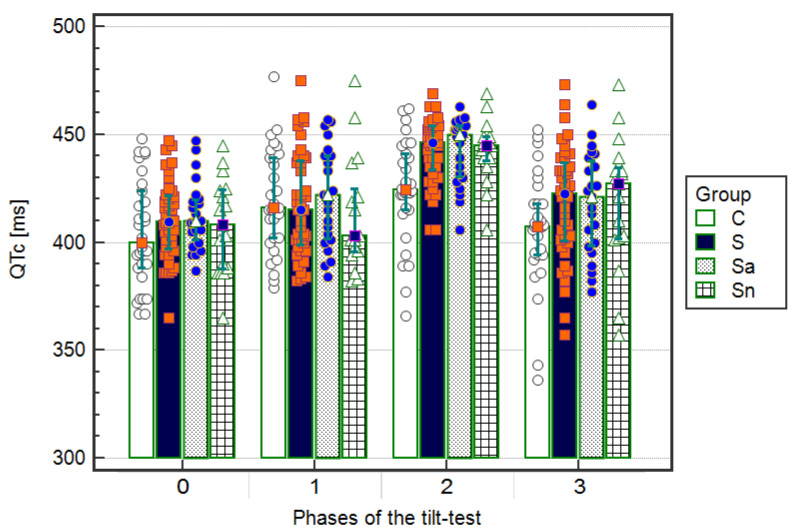
Values of QTc intervals in electrocardiograms (ECGs) on admission to the hospital (Phase 0) and during the resting supine (Phase 1), the upright phase (Phase 2), and the returning to supine phase (Phase 3) in the group: S—syncopal, C—control, Sa—syncopal with abnormal T, Sn—syncopal with normal T. Values [ms] are shown as a median and interquartile range. A significant difference (*p* < 0.05) while comparing the syncopal group to the control group was calculated using the ANOVA Friedman test with the Iman-Davenport statistic and post hoc test (Conover-Iman).

The QTc intervals, shown as median and IQR [ms], were significantly (*p* < 0.05) longer during the upright phase (QTc2) compared to ECGs during the other phases (QTc0, QTc1, QTc3) in the syncopal group: 446 (20) vs. 410 (24) and 415 (29) and 423 (30), respectively. Furthermore, after dividing into subgroups, 23 patients with an abnormal T wave: 450 (24) vs. 410 (20) and 422 (39) and 421 (41), respectively, and 17 children without changes in the T wave: 445 (10) vs. 408 (36) and 403 (25) and 427 (31), respectively, as well as in the control group, except QTc1 in the resting supine phase: 425 (26) vs. 400 (36) and 416 (31) and 407 (24), respectively. There were significant (*p* < 0.05) differences in the QTc2 intervals during the upright phase compared to the other phases (QTc0, QTc1, QTc3) between the group of syncopal children and the control group with no differences between the children with T wave morphology abnormalities and a normal T wave (Figure 3, Table 4).

**Figure 4:**
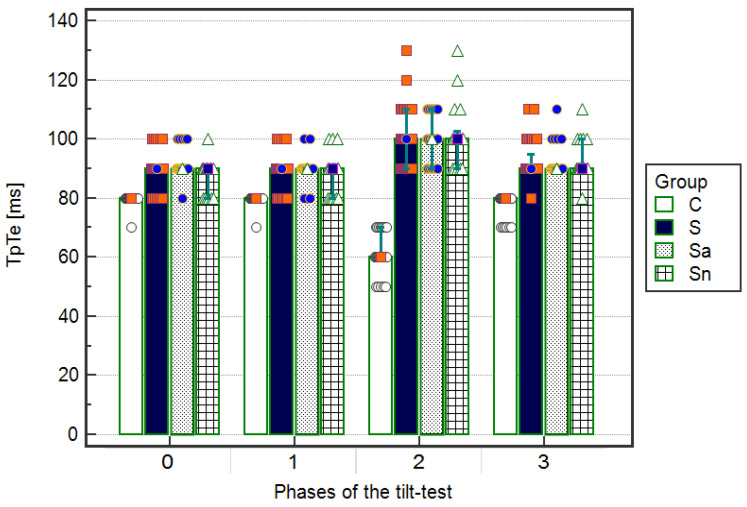
Values of TpTe intervals in ECG on admission to hospital (Phase 0), the resting supine phase (Phase 1), the upright phase (Phase 2), and the returning to supine phase (Phase 3) in the groups: S—syncopal, C—control, Sa—syncopal with abnormal T, Sn—syncopal with normal T. Values [ms] are shown as a median and interquartile range. A significant difference (*p* < 0.001) while comparing the syncopal group to the control group was calculated using the ANOVA Friedman test with the Iman-Davenport statistic and post hoc test (Conover-Iman).

The TpTe intervals, shown as median and IQR [ms] were substantially (*p* < 0.001) longer during the upright phase (TpTe2) compared to the ECGs during the other phases of the tilt test (TpTe0, TpTe1, TpTe3) in the syncopal children: 100 (20) vs. 90 (0) and 90 (0) and 90 (5), respectively. In addition, after dividing into subgroups, the abnormal T wave: 100 (20) vs. 90 (0) and 90 (0) and 9 (0), respectively, without changes in the repolarization period: 100 (10) vs. 90 (10) and 90 (10) and 90 (10), respectively. In the control group, the TpTe intervals [ms] were significantly (*p* < 0.001) shorter in the upright phase compared to the supine-related resting and returning phases: 60 (10) vs. 80 (0) and 80 (0) and 80 (0), respectively, with substantial (*p* < 0.001) differences from the syncopal group. Among the children with T wave morphology abnormalities and normal T wave morphology, significant (*p* < 0.05) differences were only seen in ECGs performed on admission to the hospital (Figure 4, Table 5).

**Figure 5:**
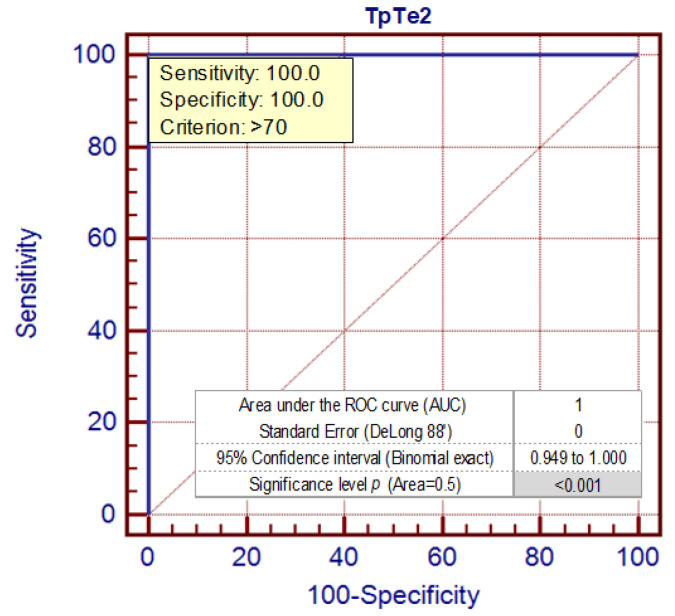
The ROC curve (AUC) analysis for TpTe intervals [ms] in a syncopal group and control group in the upright phase during the HUTT—Phase 2 (TpTe2).

The cut-off point for TpTe intervals that better optimizes the values of sensitivity (100) and specificity (100) is demonstrated for values >70 ms (Figure 5).

**Figure 6:**
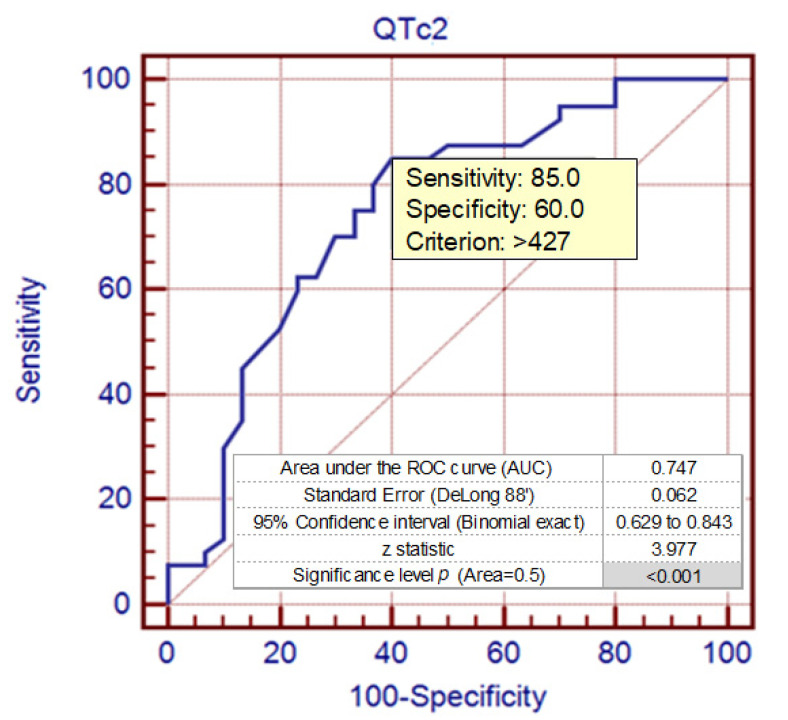
The ROC curve (AUC) analysis for QTc intervals [ms] in the syncopal group and control group in the upright phase during the HUTT—Phase 2 (TpTe2).

The cut-off point for QTc intervals that better optimizes the values of sensitivity (85.0) and specificity (60.0) is for values >427 ms (Figure 6).

**Figure 7:**
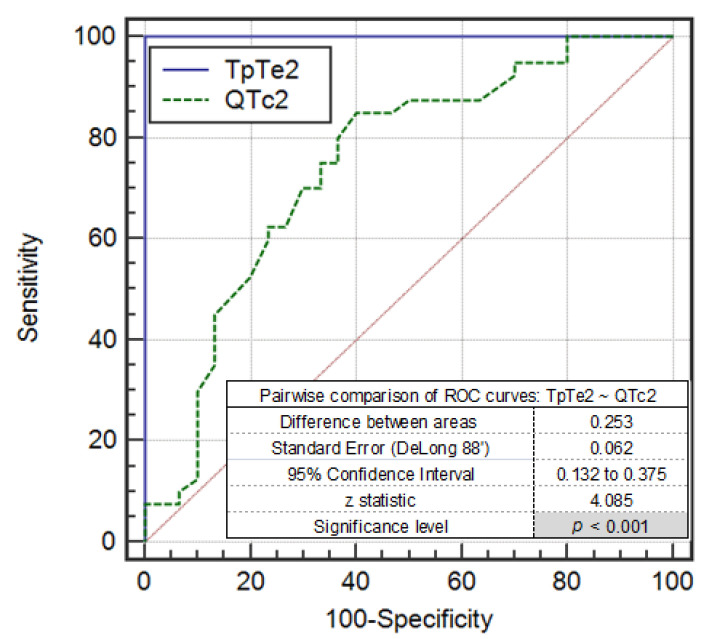
The pairwise comparison of ROC curves TpTe2~QTc2 [ms] in the syncopal group and control group in the upright phase during the HUTT—Phase 2 (TpTe2~QTc2).

The area under the ROC curve (AUC) for TpTe in the upright position was 1.00, which indicates that this variable is a very good discriminator and better than QTc (AUC = 0.747). This is also confirmed in the results for the pairwise comparison of ROC curves TpTe2~QTc2 (Figure 7).

## 4. Discussion

The main cause of syncope in children with reflex vasovagal syncope (VVS) is dysregulation between sympathetic and parasympathetic control of cardiovascular functions, which has been already detected in these children before syncope [5]. The imbalance in the sympathetic nervous system initially imputed the probable cause of abnormal electrocardiographic repolarization and ventricular arrhythmias. The indicator of cardiac autonomic dysfunction is the T-wave dispersion that can be easily demonstrated in a standard electrocardiogram (ECG) [11].

T wave parameters, QT and TpTe intervals (the last section of T-wave), reflect dispersion of repolarization. Its amplification may lead to development of ventricular arrhythmias and syncope [8,9,12]. The questions of whether the TpTe interval reflects transmural repolarization heterogeneity or total dispersion of repolarization [13,14] is still a matter of debate. Therefore, in clinical practice, the TpTe interval is considered a more sensitive marker of arrhythmogenesis and increased risk of mortality in long QT syndrome (LQTS) compared to the QT interval [7,15]. In clinical evaluation, the TpTe interval, in addition to the QT interval, has been found amplified in patients with congenital and acquired LQTS [8,13].

The study group included 40 children with a structurally normal heart, clinical characteristics of VVS, and a negative HUTT, and 30 healthy children without syncope (control group) who also presented negative results of tilt testing. In the group of 40 children, syncope was often caused by typically prolonged verticalization and/or a sudden change of body position (57.5%) and in a setting of emotional stress, fear, pain, or blood sampling (42.5%). However, 45% of children also have episodes of vasovagal syncope, which occur after exercise.

In 40% of the study children, syncope was followed by weakness and it was mostly preceded by characteristic prodromal symptoms such as dizziness, headache, breathlessness, and visual disturbances. The presence of preceding palpitations (in 22% of children) and chest pain (15%) was also not typical of vasovagal syncope and it can manifest symptoms of arrhythmias.

Similarly, Thilenius et al. described children with repeated syncopal episodes and presyncopes in the upright position, which were preceded by dizziness or loss of vision without loss of muscle tone. In some patients, episodes also occurred in relation to physical exertion. However, all these children experienced syncope with systemic hypotension and bradycardia during head-up tilt testing [16]. In our study, the tilt tests were negative without reflex hypotension and/or bradycardia.

Syncope is the most common initial symptom (69%) in patients with congenital LQTS with a predisposition to malignant ventricular tachyarrhythmias (torsade de pointes), which potentially lead to recurrent syncope and sudden cardiac death (SCD) [13,17].

In contrast to the benign course of VVS, syncopal episodes in LQTS are associated with high mortality [18]. Exertional syncope that mainly occurs during swimming or in response to auditory or emotional triggers should result in a high index of suspicion for a cardiac etiology. In particular, it has been associated with LQTS and catecholaminergic polymorphic ventricular tachycardia (CPVT) [6,17]. In addition, risk factors that raise suspicion of a cardiac etiology include the absence of prodromal symptoms, the presence of preceding palpitations within seconds of loss of consciousness, a family history of sudden cardiac death, abnormal physical examination, and abnormal ECG [6,17].

In the present study, none of the children had a structural heart disease or a positive family history. However, 45% of the patients have exertional syncope, preceding palpitations and chest pain (37%) that might suggest an arrhythmogenic cause of syncope. However, we did not find ventricular arrhythmia in these children in ECGs and during Holter monitoring.

Head-up tilt testing (HUTT) with the upright phase was useful for identifying repolarization abnormalities with prolongation of electrocardiographic T wave parameters independently of the changes in the heart rate in response to sympathetic stimulation, which is related to LQTS children [18,19,20]. 

The HUTT can also be used to identify patients with the long QT syndrome (LQTS) among patients with the VVS syncope [13].

Similarly, abnormalities of T wave morphology with an inadequate adaptation of the QT, TpTe intervals to changes in the RR interval by sympathetic stimulation were observed in children with LQTS during the exercise test. These findings may be useful in directing genetic testing [21,22]. 

In healthy children, during sympathetic stimulation, the repolarization parameters (QT and, especially, TpTe interval) shorten in response to acceleration of the heart rhythm during brief tachycardia without abnormal T wave morphology [22].

In the present study, during the abort upright phase of HUTT, the children with syncope and the control subjects presented tachycardia when stimulation of the sympathetic system occurred with a similar heart rate (RR interval) in both groups. However, the response of the QT interval to this tachycardia differed. Since the RR interval shortened more than the QT interval during tachycardia, the corrected QT (QTc) increased more in the syncopal group than in the control group (*p* < 0.001). In children with syncope, this may result in a large variability of TpTe and QT parameters for simple autonomic maneuvers that invoke reflex tachycardia, such as brisk standing [23,24].

Similarly, the response of QT interval to brief tachycardia was particularly impaired in the children with LQTS. In 2010, Viskin et al. showed that prolongation of the QT interval induced by brisk standing could aid diagnosis of a long QT syndrome [25].

Additionally, the TpTe interval was significantly longer in the children with syncope (like in the children with LQTS) in contrast to the healthy group whose TpTe was shorter during the upright phase compared to the ECGs on admission to the hospital as well as the other phases of the tilt test. Similarly, a study by Butta et al. demonstrated prolongation of the QTc and TpTe intervals in the adult patients with syncope compared to control subjects. Moreover, the values of QTc > 424.8 ms and TpTe > 100 ms had a predictive value for diagnosis of syncope [24].

HUTT is used as an aid in establishing the diagnosis of VVS and may be a useful tool in evaluating syncopal children for diagnosing abnormal T wave parameters and predisposition to ventricular arrhythmias [23,24,26].

Based on the morphology and amplitude of the T-wave in the ECG in the upright phase, among the group with VVS, we detected 23 children with a bifid or flat T wave and 17 children with a normal T wave without significant differences in the QTc and TpTe intervals between these groups during the phases of tilt testing.

Similarly, Mayuga et al. described very flat T waves in 39% of patients and negative T waves in 21% of subjects with VVS in the ECG V5 during the upright phase of a negative HUTT [26].

In particular, the electrocardiographic bifid T waves are typical of the long QT syndrome (66% to 88% of patients with type 2 LQTS) and correlate well with increased arrhythmic risk [13,21].

In available literature, the patients with syncope and negative HUTT (without development of reflex hypotension and/or bradycardia) were presented as control groups compared to the groups of children with VVS and a positive result of HUTT. In our recent study, we compared a smaller group of children with VVS to the control group of children diagnosed with psychogenic pseudosyncope (PPS), and we suggested necessary further studies. Additionally, in larger groups, this established the clinical importance of abnormal dynamics of the T wave parameters in these children [27]. In the present study, among 40 children with VVS and a negative HUTT, 23 patients with bifid and/or flat T-wave and abnormal prolonged values of QTc and TpTe intervals, with a greater predisposition to ventricular arrhythmias, were identified. These patients were not treated as a control group. Therefore, the control group consisted of 30 healthy volunteers without syncope and with negative results of HUTT. The head-up tilt test is not usually performed in healthy children, but our patients and their parents gave their consent to this procedure. Our children, aged 14–17, tolerated long standing upright position very well while performing HUTT.

These are novel findings in children with VVS and no similar data have been found in available publications to date.

The number of our study participants with VVS is very limited and in the literature reports [2,3], similarly to our VVS study group, VVS occurs more frequently in girls (27/40). In addition, we found it difficult to include a greater number of female volunteers in the control group (12/30). More boys were willing to participate since they are braver. The differences in sex distribution between the syncopal and control groups should not affect the final results. In our recent study [22], in the large group of 320 healthy children, we did not find significant differences in QTc (*p* = 0.242) and TpTe (*p* = 0.220) intervals between the girls and boys.

The current 2018 guidelines of the European Society of Cardiology (ESC) and the European Heart Rhythm Association (EHRA) state that assessment of the patients with syncope should begin with a careful medical history, physical examination, and electrocardiogram (ECG), which can lead to a diagnosis of syncope in more than 50% to 60% of patients [3].

In our study, we concluded that 12-lead standard ECG during tilt table testing remains the primary, cheap, and most commonly used cardiac diagnostic tool that can help identify affected children with vasovagal syncope, which may have implications for management and prevention, according to 2015 ESC guidelines (for the management of patients with ventricular arrhythmias and the prevention of sudden cardiac death) [28,29].

Prolongation of electrocardiographic TpTe and QTc parameters, with the abnormal T wave morphology (bifid and/or flat T wave), measured during the upright tilt phase of the HUTT, could be considered as possible independent markers of syncope severity, according to arrhythmic potential, which requires careful follow-up with possible long-term ECG recordings and genetic testing in the future.

## 5. Conclusions

Prolongation of electrocardiographic TpTe and QT intervals, in addition to the abnormal T wave morphology recorded during the HUTT, are helpful for identifying VVS children more predisposed to ventricular arrhythmias with a latent risk of LQTS. Further studies are required to assess the values of these repolarization parameters in clinical practice, particularly in even larger patient groups with smaller differences in sex distribution.

## Figures and Tables

**Table 1:** Clinical characteristics of children with vasovagal syncope (VVS) (Syncopal group).

Categories	Syncopal Group (*n* = 40)
Age (years) *	15.8 (2.3)
Female, *n* (%)	27 (68%)
*Circumstances of syncope*	***n*** **(%)**
Prolonged verticalization	23 (57.5%)
Sudden change of body position	23 (57.5%)
Emotional stress (fear, pain)	16 (40%)
Post-exercise	18 (45%)
*Prodromal symptoms*	***n*** **(%)**
Chest pain	6 (15%)
Palpitations	9 (22.5%)
Dizziness	14 (35%)
Breathlessness	12 (30%)
Sweating	5 (2%)
Vision disorder	11 (27.5%)
Tinnitus	4 (10%)
Pale skin	3 (7.50%)
Tremors in the limbs	6 (15%)
Weakness	16 (40%)
Headache	15 (37.5%)
Hot feeling	1 (2.5%)
*The period of syncope before hospitalization*	***n*** **(%)**
Occurs for 2–4 years	10 (25%)
Occurs in 1 year	11 (27.5%)
Up to 6 months	9 (22.5%)
Up to 3 months	10 (25%)
*Experience of syncope*	***n*** **(%)**
at least 3 to 4 times in the year	19 (47%)
1–2 times in a month	14 (35%)
1–2 times in a week	7 (17.5%)

* Median and interquartile range (IQR).

**Table 2:** Comparison of electrocardiographic parameters during the head-up tilt table test (HUTT) in the children diagnosed with vasovagal syncope (VVS) (Syncopal group) and healthy volunteers without syncope (Control group).

ECG Parameters * [ms]	Syncopal Group (*n* = 40)		Control Group (*n* = 30)		*p* Value
	Median	IQR	Median	IQR	
RR0	825	205	865	190	0.606
QT0	370	30	370	30	0.735
QTc0	410	24	400	36	0.260
TpTe0	90	0	80	0	**<0.001**
RR1	815	190	810	230	0.767
QT1	390	30	375	30	0.713
QTc1	415	29	416	37	0.687
TpTe1	89	0	80	0	**<0.001**
RR2	605	95	640	120	0.088
QT2	340	25	340	20	0.310
QTc2	446	20	425	26	<0.001
TpTe2	100	20	60	10	**<0.001**
RR3	750	185	740	210	0.541
QT3	360	30	360	30	0.767
QTc3	423	30	407	24	0.090
TpTe3	90	5	80	0	**<0.001**

* Values of intervals in ECG during the tilt table test (HUTT) on admission to hospital (RR0, QT0, QTc0, TpTe0) and during the rest phase (RR1, QT1, QTc1, TpTe1), the upright 2 phases (RR2, QT2, QTc2, TpTe2), and recovery phase (RR3, QT3, QTc3, TpTe3). Values [ms] are shown as a median and interquartile range (IQR). QTc intervals corrected for the heart rate (HR) using the Bazett’s formula. A significant difference (*p* < 0.05) (shown in bold) while comparing Syncopal group to the Control group was calculated using the Mann-Whitney U test with the Marascuillo and McSweeney continuity correction.

**Table 3:** Inter-time comparison of RR intervals in ECGs on admission to the hospital and during three phases of HUTT.

*p* Value **	Group C	Group S	Group Sa	Group Sn
RR0 vs. RR1	**0.069**	0.949	0.868	0.775
RR0 vs. RR2	**<0.001**	**<0.001**	**<0.001**	**<0.001**
RR0 vs. RR3	**0.014**	**0.002**	**0.040**	**0.026**
RR1 vs. RR2	**<0.001**	**<0.001**	**<0.001**	**<0.001**
RR1 vs. RR3	0.513	**0.002**	0.059	**0.013**
RR2 vs. RR3	**<0.001**	**<0.001**	**<0.001**	**<0.001**
*p* Value *	**<0.001**	**<0.001**	**<0.001**	**<0.001**

Phase 0 (RR0)—on admission to the hospital, Phase 1 (RR1)—during the resting supine phase, Phase 2 (RR2)—the upright phase and Phase 3 (RR3)-the returning to supine phase—in the groups: C—control, S—syncopal, Sa—syncopal with abnormal T, Sn—syncopal with normal T. Values [ms] are shown as a median and interquartile range. A significant difference (*p* < 0.05) (shown in bold) was calculated using the ANOVA Friedman test with the Iman-Davenport statistic (*p* *) and post hoc test (*p* **) (Conover-Iman).

**Table 4:** Inter-time comparison of QTc intervals in ECGs on admission to hospital and during three phases of HUTT.

*p* Value **	Group C	Group S	Group Sa	Group Sn
QTc0 vs. QTc1	**0.004**	**0.013**	**0.025**	0.247
QTc0 vs. QTc2	**<0.001**	**<0.001**	**<0.001**	**<0.001**
QTc0 vs. QTc3	0.552	**0.026**	0.119	0.118
QTc1 vs. QTc2	0.627	**<0.001**	**<0.001**	**<0.001**
QTc1 vs. QTc3	**0.022**	0.784	0.475	0.677
QTc2 vs. QTc3	**0.006**	**<0.001**	**<0.001**	**<0.001**
*p* Value *	**<0.001**	**<0.001**	**<0.001**	**<0.001**

Phase 0 (RR0)-on admission to the hospital, Phase 1 (QTc1)—during the resting supine phase, Phase 2 (QTc2)—the upright phase and Phase 3 (QTc3)—the returning to supine phase—in the groups: C—control, S—syncopal, Sa—syncopal with abnormal T, Sn—syncopal with normal T. Values [ms] are shown as a median and interquartile range. A significant difference (*p* < 0.05) (shown in bold) was calculated using the ANOVA Friedman test with the Iman-Davenport statistic (*p* *) and post hoc test (*p* **) (Conover-Iman).

**Table 5:** Inter-time comparison of TpTe intervals in ECGs on admission to hospital and during three phases of HUTT.

*p* Value **	Group C	Group S	Group Sa	Group Sn
TpTe0 vs. TpTe1	1.000	0.621	0.172	0.348
TpTe0 vs. TpTe2	**<0.001**	**<0.001**	**<0.001**	**<0.001**
TpTe0 vs. TpTe3	**<0.001**	**0.012**	0.527	**0.001**
TpTe1 vs. TpTe2	**<0.001**	**<0.001**	**<0.001**	**<0.001**
TpTe1 vs. TpTe3	**<0.001**	**0.003**	0.048	**0.019**
TpTe2 vs. TpTe3	**<0.001**	**<0.001**	**<0.001**	**<0.001**
*p* Value *	**<0.001**	**<0.001**	**<0.001**	**<0.001**

Phase 0 (TpTe0)—on admission to the hospital, Phase 1 (TpTe1)—during the resting supine phase, Phase 2 (TpTe2)—the upright phase and Phase 3 (TpTe3)—the returning to supine phase—in the groups: C—control, S—syncopal, Sa—syncopal with abnormal T, Sn—syncopal with normal T. Values [ms] are shown as a median and interquartile range. A significant difference (*p* < 0.05) (shown in bold) was calculated using the ANOVA Friedman test with the Iman-Davenport statistic (*p* *) and post hoc test (*p* **) (Conover-Iman).

## References

[1] Moya A., Sutton R., Brignole M., Ammirati F., Blanc J.J., Dahm J.B., Deharo J.C., Gajek J., Gjesdal K., Krahn A. (2009). Guidelines for the diagnosis and management of syncope. Eur. Heart J..

[2] Wieling W., Ganzeboom K.S., Saul J.P. (2004). Reflex syncope in children and adolescents. Heart.

[3] Brignole M., Moya A., de Lange F.J., Deharo J.C., Elliott P.M., Fanciulli A., Furlan R., Kenny R.A., Martín A., Probst V. (2018). 2018 ESC Guidelines for the diagnosis and management of syncope. Eur. Heart J..

[4] Brignole M., Menozzi C., Del Rosso A., Costa S., Gaggioli G., Bottoni N.A., Bartoli P., Sutton R. (2000). New classification of haemodynamics of vasovagal syncope: Beyond the VASIS classification. Europace.

[5] Rogers C., O’Flynn N. (2011). NICE guideline: Transient loss of consciousness (blackouts) in adults and young people. Br. J. Gen. Pract..

[6] Priori S.G., Blomström-Lundqvist C., Mazzanti A., B loma N., Borggrefe M., Camm J., Elliott P.M., Fitzsimons D., Hatala R., Hindricks G. (2015). 2015 ESC Guidelines for the management of patients with ventricular arrhythmias and the prevention of sudden cardiac death. Eur. Heart J..

[7] Antzelevitch C., Di Diego J.M. (2019). Tpeak-Tend interval as a marker of arrhythmic risk. Heart Rhythm.

[8] Yamaguchi M., Shimizu M., Ino H., Terai H., Uchiyama K., Oe K., Mabuchi T., Konno T., Kaneda T., Mabuchi H. (2003). T wave peak-to-end interval and QT dispersion in acquired long QT syndrome: A new index for arrythmogenicity. Clin. Sci..

[9] Watanabe N., Kobayashi Y., Tanno K., Miyoshi F., Asano T., Kawamura M., Mikami Y., Adachi T., Ryu S., Miyata A. (2004). Transmural dispersion of repolarization and ventricular tachyarrhythmias. J. Electrocardiol..

[10] Rautaharju P.M., Surawicz B., Gettes L.S. (2009). AHA/ACCF/HRS recommendations for the standardization and interpretation of the electrocardiogram: Part IV: The ST segment, T and U waves, and the QT interval: A scientific statement from the American Heart Association Electrocardiography and Arrhythmias Committee, Council on Clinical Cardiology; the American College of Cardiology Foundation; and the Heart Rhythm Society; endorsed by the International Society for Computerized Electrocardiology. Circulation.

[11] Sucu M., Ozer O., Davutoglu V., Ercan S., Yuce M., Coskun F.Y. (2015). Relationship between neurocardiogenic syncope and ventricular repolarization. Pace.

[12] Amoozgar H., Hosseiniasl M. (2012). T-peak to T-end abnormality in pediatric patients with syncope. Iran. J. Pediatr..

[13] Zhang L., Timothy K.W., Vincent G.M., Lehmann M.H., Fox J., Giuli L.C., Shen J., Splawski I., Priori S.G., Compton S.J. (2000). Spectrum of ST-T-wave patterns and repolarization parameters in congenital long-QT syndrome. ECG findings identify genotype. Circulation.

[14] Opthof T., Coronel R., Wilms-Schopman F.J.G., Plotnicov A.N., Shlapakova I.N., Danilo P., Rosen M.R., Janse M.J. (2007). Dispersion of repolarization in canine ventricle and the electrocardiographic T wave: Tp-e interval does not reflect transmural dispersion. Heart Rhythm.

[15] Markiewicz-Łoskot G., Moritz-Janiszewska E., Mazurek B., Łoskot M., Bartusek M., Skierska A., Szydłowski L. (2018). Electrocardiographic T-wave parameters in families with long QT syndrome. Adv. Clin. Exp. Med..

[16] Thilenius O.G., Quinones J.A., Husayni T.S., Novak J. (1991). Tilt Test for Diagnosis of Unexplained Syncope in Pediatric Patients. Pediatrics.

[17] Mac Cormick J.M., Crawford J.R., Chung S.-K., Shelling A.N., Evans C.A., Rees M.I., Smith W.M., Crozier I.G., McAlister H., Skinner J.R. (2011). Symptoms and signs associated with syncope in young people with primary cardiac arrhythmias. Heart Lung Circ..

[18] Toft E., Aaroe J., Jensen B.T., Christiansen M., Fog L., Thomsen P.E.B., Kanters J.K. (2003). Long QT syndrome patients may faint due to neurocardiogenic syncope. Europace.

[19] Hermosillo A.G., Falcón J.C., Márquez M.F., Arteaga D., Cárdenas M. (1999). Positive head-up tilt table test in patients with the long QT syndrome. Europace.

[20] Ogawa Y., Aiba T., Kamei N., Tominaga K., Fujita H., Miyamoto Y., Tanaka T., Kido S. (2016). Coexistence of congenital long QT syndrome and autonomic deregulation in children. Pediatrics Int..

[21] Takenaka K., Tomohiko A., Shimizu W., Kobori A., Ninomiya T., Otani H., Kubota T., Takaki H., Kamakura S., Horie M. (2003). Exercise stress test amplifies genotype-phenotype correlation in the LQT1 and LQT2 forms of the long-QT syndrome. Circulation.

[22] Markiewicz-Łoskot G. (2018). Electrocardiographic characteristics of a total of repolarization (QT), early repolarization phase (QTP) and late phase repolarization (TpTe) in healthy children and children with long QT syndrome. Adv. Clin. Exp. Med..

[23] Medow M.S., Merchant S., Suggs M., Terilli C., O’Donnell-Smith B., Stewart J.M. (2018). Postural Heart Rate Changes with Vasovagal Syncope. Pediatrics.

[24] Buttà C., Tuttolomondo A., Casuccio A., Di Raimondo D., Giarrusso L., Miceli G., Lo Vecchio S., Canino B., Licata G., Pinto A. (2014). Use of QT intervals for a more accurate diagnose of syncope and evaluation of syncope severity. Int. J. Clin. Pract..

[25] Viskin S., Postema P.G., Bhuiyan Z.A., Rosso R., Kalman J.M., Vohra J.K., Guevara-Valdivia M.E., Marquez M.F., Kogan E., Belhassen B. (2010). The Response of the QT Interval to the Brief Tachycardia Provoked by Standing: A Bedside Test for Diagnosing Long QT Syndrome. J. Am. Coll. Cardiol..

[26] Mayuga K.A., Fouad-Tarazi F. (2007). Dynamic changes in T-wave amplitude during tilt table testing: Correlation with outcomes. Ann. Noninvasive Electrocardiol..

[27] Kolarczyk E., Markiewicz-Łoskot G., Szydłowski L. (2020). The repolarization period during the head-up tilt test in children with vasovagal syncope. Int. J. Environ. Res. Public Health.

[28] Van Camp G., Pasquet A., Sinnaeve P., Mairesse G.H., De Pauw M., Claeys M.J. (2016). Summary 2015 ESC guidelines. Acta Cardiol..

[29] Markiewicz-Łoskot G., Moric-Janiszewska E., Mazurek U. (2009). The risk of cardiac events and management of LQTS patients on the basis of genotype. Ann. Noninvasive Electrocardiol..

